# Supplementary material for: The Small RNA Universe of Capitella teleta
Source: Front Mol Biosci. 2022 Feb 25;9:802814. doi: 10.3389/fmolb.2022.802814 (PMC8915122; doi:10.3389/fmolb.2022.802814)
Supplement: Supplementary file 1 [file DataSheet1.ZIP › Supplement/known/CAPTEscaffold_610_25957.pdf]

Provisional ID	:	CAPTEscaffold_610_25957
Score total	:	612760
Score for star read(s)	:	3.9
Score for read counts	:	612749.3
Score for mfe	:	2.3
Score for randfold	:	1.6
Score for cons. seed	:	3
Total read count	:	1201893
Mature read count	:	1201561
Loop read count	:	0
Star read count	:	332

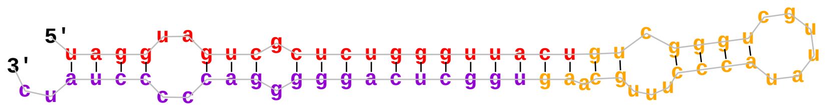

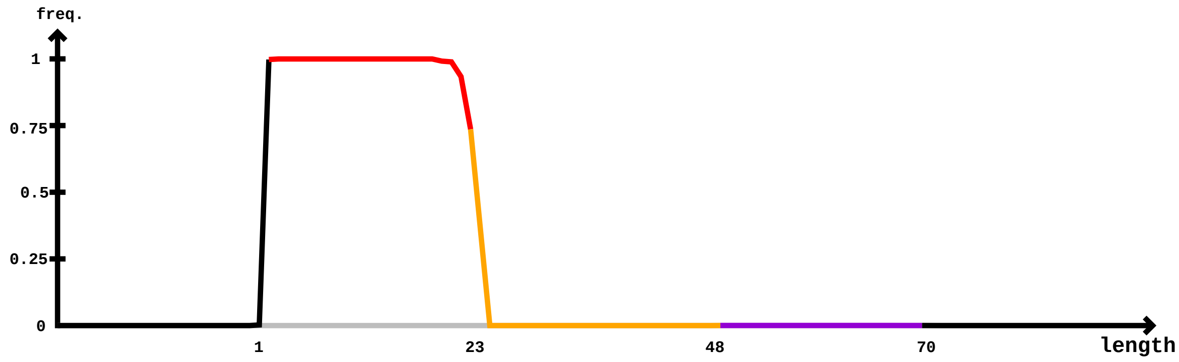

Mature Star

```
5'- uguugcgcgggacccuucgauagguagucgcucuggguuacugucgggucguuauacccuuugcaaguggcucagggggacccccuauccaagggcugcuugaucgcuacgu   -3'   obs
    uguugcgcgggacccuucgauagguagucgcucuggguuacugucgggucguuauacccuuugcaaguggcucagggggacccccuauccaagggcugcuugaucgcuacgu         exp
    uguugcgcgggacccuucgauagguagucgcucuggguuacugucgggucguuauacccuuugcaaguggcucagggggacccccuauccaagggcugcuugaucgcuacgu         known
    .((.(((((((.(((((.((((((..(((.((((((((((((((.((((......))))...)).)))))))))))).)))..)))))).)))))...))))..))).))..   reads   mm   sample

    ..................Aauagguagucgcucuggguuacu......................................................................   1   1   seq
    ...................auagguagucgcucuAgguu.........................................................................   1   1   seq
    ...................auagguagucgcucuggguu.........................................................................   86   0   seq
    ...................auagguagucgcucuggguua........................................................................   139   0   seq
    ...................auagguagucgcucuggguuU........................................................................   1   1   seq
    ...................auagguaguAgcucuggguuac.......................................................................   1   1   seq
    ...................auagguagucgcucugUguuac.......................................................................   1   1   seq
    ...................auagguagucgcucuggguuaU.......................................................................   7   1   seq
    ...................auagguagucgcucuggguuUc.......................................................................   1   1   seq
    ...................auUgguagucgcucuggguuac.......................................................................   2   1   seq
    ...................auagguagucgcucugggCuac.......................................................................   1   1   seq
    ...................Cuagguagucgcucuggguuac.......................................................................   90   1   seq
    ...................Guagguagucgcucuggguuac.......................................................................   8   1   seq
    ...................auagguagucgcucuggguuac.......................................................................   1543   0   seq
    ...................auagguagucAcucuggguuac.......................................................................   1   1   seq
    ...................Nuagguagucgcucuggguuac.......................................................................   1   1   seq
    ...................auagguagucgcucuggguuacu......................................................................   576   0   seq
    ...................Guagguagucgcucuggguuacu......................................................................   1   1   seq
    ...................auagguaUucgcucuggguuacu......................................................................   1   1   seq
    ...................Cuagguagucgcucuggguuacu......................................................................   25   1   seq
    ...................auagguagucgAucuggguuacu......................................................................   1   1   seq
    ...................auagguagucgcucuggguuacC......................................................................   303   1   seq
    ...................auagguagucgcucugggCuacu......................................................................   1   1   seq
    ...................auagguagucgcucAggguuacu......................................................................   1   1   seq
    ...................auagguagAcgcucuggguuacu......................................................................   1   1   seq
    ...................auagguagucgcucuggguuaUu......................................................................   88   1   seq
    ...................Uuagguagucgcucuggguuacu......................................................................   4   1   seq
    ...................auagguagucgcGcuggguuacu......................................................................   1   1   seq
    ...................auagguagucgcucuggguuacuU.....................................................................   4   1   seq
    ....................uagguagAcgcucugggu..........................................................................   1   1   seq
    ....................uaggGagucgcucugggu..........................................................................   1   1   seq
```

Mature **Star**

```
uguugcgcgggacccuucgauagguagucgcucuggguuacugucgggucguuauacccuuugcaaguggcucagggggacccccuauccaagggcugcuugaucgcuacgu
....................uGgguagucgcucugggu..........................................................................  51     1  seq
....................uagguagucgcucugggG..........................................................................  44     1  seq
....................uNgguagucgcucugggu..........................................................................  1      1  seq
....................Aagguagucgcucugggu..........................................................................  12     1  seq
....................uagguagucgcucugggA..........................................................................  4584   1  seq
....................Cagguagucgcucugggu..........................................................................  1      1  seq
....................uaggAagucgcucugggu..........................................................................  1      1  seq
....................uaUguagucgcucugggu..........................................................................  1      1  seq
....................uagguagucgcucuAggu..........................................................................  6      1  seq
....................uaAguagucgcucugggu..........................................................................  3      1  seq
....................Gagguagucgcucugggu..........................................................................  8      1  seq
....................uagguUgucgcucugggu..........................................................................  2      1  seq
....................uagguaguUgcucugggu..........................................................................  2      1  seq
....................uagguagucgcucugggu..........................................................................  4988   0  seq
....................uagguagucgcucugAgu..........................................................................  1      1  seq
....................uagguagucgcuUugggu..........................................................................  1      1  seq
....................uagguagucgcucuCggu..........................................................................  1      1  seq
....................uagguagucgcucuggAu..........................................................................  2      1  seq
....................uagguagucgAucugggu..........................................................................  2      1  seq
....................uagguaguAgcucuggguu.........................................................................  2      1  seq
....................uagguagucgcucuggguA.........................................................................  4      1  seq
....................uagAuagucgcucuggguu.........................................................................  2      1  seq
....................uagguagucgcucuggguG.........................................................................  1      1  seq
....................Gagguagucgcucuggguu.........................................................................  4      1  seq
....................uagguagucgcuGuggguu.........................................................................  1      1  seq
....................Nagguagucgcucuggguu.........................................................................  2      1  seq
....................uagguagucgcucuCgguu.........................................................................  1      1  seq
....................Aagguagucgcucuggguu.........................................................................  12     1  seq
....................uagguagucgcucuAgguu.........................................................................  6      1  seq
....................uagguagucgcuAuggguu.........................................................................  2      1  seq
....................uagguagucgcucuggguu.........................................................................  3254   0  seq
....................uagguaguGgcucuggguu.........................................................................  1      1  seq
....................uaggGagucgcucuggguu.........................................................................  1      1  seq
....................uagguagucgcucugAguu.........................................................................  1      1  seq
....................uagUuagucgcucuggguu.........................................................................  1      1  seq
....................uaCguagucgcucuggguu.........................................................................  1      1  seq
....................uagguagucgcucAggguu.........................................................................  1      1  seq
....................uagguGgucgcucuggguu.........................................................................  1      1  seq
....................uagAuagucgcucuggguua........................................................................  5      1  seq
....................uagguagucgcucuggguua........................................................................  64186  0  seq
....................uagguaguAgcucuggguua........................................................................  22     1  seq
....................uagguagucgGucuggguua........................................................................  3      1  seq
....................uaAguagucgcucuggguua........................................................................  8      1  seq
....................uagguagucgcucuggguAa........................................................................  9      1  seq
....................uagguagucgcucugCguua........................................................................  2      1  seq
....................uagguCgucgcucuggguua........................................................................  1      1  seq
....................uagguagAcgcucuggguua........................................................................  21     1  seq
....................uagguagucgcAcuggguua........................................................................  3      1  seq
....................uagguagucgcucCgggua.........................................................................  14     1  seq
....................uagguagucgcucuggguGa........................................................................  3      1  seq
....................uagCuagucgcucuggguua........................................................................  4      1  seq
....................uagguagGcgcucuggguua........................................................................  1      1  seq
....................uagguagucgcucuggAuua........................................................................  9      1  seq
....................uaggGagucgcucuggguua........................................................................  7      1  seq
....................uagguagucgcucGggguua........................................................................  1      1  seq
....................uagguagucgcucugggGua........................................................................  4      1  seq
....................uagguagucgcucAggguua........................................................................  11     1  seq
....................uagguagucgcucuggCuua........................................................................  3      1  seq
....................uagguaAucgcucuggguua........................................................................  6      1  seq
....................uagguagucgcuGuggguua........................................................................  1      1  seq
....................uagguagucgcucuUgguua........................................................................  6      1  seq
....................Gagguagucgcucuggguua........................................................................  85     1  seq
....................uaUguagucgcucuggguua........................................................................  4      1  seq
....................uagguaguUgcucuggguua........................................................................  16     1  seq
....................uagUuagucgcucuggguua........................................................................  2      1  seq
....................uGgguagucgcucuggguua........................................................................  7      1  seq
....................uagguagucgcucugUguua........................................................................  4      1  seq
....................uagguagCcgcucuggguua........................................................................  1      1  seq
....................uagguaCucgcucuggguua........................................................................  4      1  seq
....................uagguagucgcGcuggguua........................................................................  19     1  seq
```

```
                    Mature                                          Star
uguugcgcgggacccuucgauagguagucgcucuggguuacugucgggucguuauacccuuugcaaguggcucagggggacccccuauccaagggcugcuugaucgcuacgu
....................uagguagucgcucuggguuC........................................................................ 4       1   seq
....................uagguagucgcucugggAua........................................................................ 17      1   seq
....................uagguaUucgcucuggguua........................................................................ 3       1   seq
....................uagguagucgcucuggguuU........................................................................ 1161    1   seq
....................uagguagucUcucuggguua........................................................................ 7       1   seq
....................Nagguagucgcucuggguua........................................................................ 15      1   seq
....................uaggCagucgcucuggguua........................................................................ 5       1   seq
....................uagguGgucgcucuggguua........................................................................ 4       1   seq
....................uaCguagucgcucuggguua........................................................................ 4       1   seq
....................uagguagucgcucuggUuua........................................................................ 7       1   seq
....................uagguUgucgcucuggguua........................................................................ 4       1   seq
....................uagguagucgUucuggguua........................................................................ 3       1   seq
....................uaNguagucgcucuggguua........................................................................ 1       1   seq
....................uagguagucgcucuggguCa........................................................................ 8       1   seq
....................uaggAagucgcucuggguua........................................................................ 31      1   seq
....................uagguagucCcucuggguua........................................................................ 5       1   seq
....................Cagguagucgcucuggguua........................................................................ 8       1   seq
....................uagguagucgcuUuggguua........................................................................ 14      1   seq
....................uagguaguGgcucuggguua........................................................................ 4       1   seq
....................uagguNgucgcucuggguua........................................................................ 1       1   seq
....................uUgguagucgcucuggguua........................................................................ 3       1   seq
....................uagguagucAcucuggguua........................................................................ 12      1   seq
....................Aagguagucgcucuggguua........................................................................ 244     1   seq
....................uagguagucgcucugggCua........................................................................ 2       1   seq
....................uagguagucgcucuCgguua........................................................................ 5       1   seq
....................uagguagucgcuAuggguua........................................................................ 16      1   seq
....................uagguagucgcCcuggguua........................................................................ 6       1   seq
....................uCgguagucgcucuggguua........................................................................ 1       1   seq
....................uagguagucgcucugAguua........................................................................ 27      1   seq
....................uagguagucgcucuggguuG........................................................................ 20      1   seq
....................uagguagucgAucuggguua........................................................................ 7       1   seq
....................uagguagucgcucuAgguua........................................................................ 77      1   seq
....................uaggGagucgcucuggguuac....................................................................... 28      1   seq
....................uagguagucgcCcuggguuac....................................................................... 20      1   seq
....................uagguagucgcucugAguuac....................................................................... 98      1   seq
....................uagguagucgcucuggguuaA....................................................................... 1503    1   seq
....................uagguCgucgcucuggguuac....................................................................... 2       1   seq
....................uagguagucgcucGggguuac....................................................................... 3       1   seq
....................uagguagucgcuUuggguuac....................................................................... 33      1   seq
....................uagguagucgcuAuggguuac....................................................................... 72      1   seq
....................uUgguagucgcucuggguuac....................................................................... 12      1   seq
....................uagguaAucgcucuggguuac....................................................................... 21      1   seq
....................uagguagucgcucuggguuCc....................................................................... 4       1   seq
....................uagguagucgcucuggguuNc....................................................................... 1       1   seq
....................uagguagucgcucuggUuuac....................................................................... 20      1   seq
....................uagguUgucgcucuggguuac....................................................................... 22      1   seq
....................uagCuagucgcucuggguuac....................................................................... 12      1   seq
....................uagguagucgcucugggCuac....................................................................... 24      1   seq
....................uagguagucgcucuggguGac....................................................................... 2       1   seq
....................uagguagucgcGcuggguuac....................................................................... 14      1   seq
....................uNgguagucgcucuggguuac....................................................................... 7       1   seq
....................uagguagucgcucuCgguuac....................................................................... 13      1   seq
....................uaggAagucgcucuggguuac....................................................................... 109     1   seq
....................uagguagucgcucuggguuaG....................................................................... 7       1   seq
....................uaUguagucgcucuggguuac....................................................................... 18      1   seq
....................uagguagucgcucuggguCac....................................................................... 17      1   seq
....................uagguGgucgcucuggguuac....................................................................... 25      1   seq
....................uagguagucgcucuggguuac....................................................................... 229429  0   seq
....................uagguagGcgcucuggguuac....................................................................... 5       1   seq
....................uagguaUucgcucuggguuac....................................................................... 23      1   seq
....................uagguagucgcucuggguuaU....................................................................... 1331    1   seq
....................uCgguagucgcucuggguuac....................................................................... 2       1   seq
....................uGgguagucgcucuggguuac....................................................................... 129     1   seq
....................uagguaNucgcucuggguuac....................................................................... 1       1   seq
....................uagguagucgcucugCguuac....................................................................... 14      1   seq
....................uagguaguGgcucuggguuac....................................................................... 26      1   seq
....................uagguagucgcucuggguAac....................................................................... 24      1   seq
....................uagguagucNcucuggguuac....................................................................... 1       1   seq
....................uagguaCucgcucuggguuac....................................................................... 12      1   seq
....................uagguagucgcAcuggguuac....................................................................... 35      1   seq
```

Mature Star

```
uguugcgcgggacccuucggauagguagucgcucuggguuacugucgggucguuauacccuuugcaaguggcucagggggacccccuauccaagggcugcuugaucgcuacgu
.....................uagguagucgcucuggguuUc.......................................................................  35      1  seq
.....................uagguagucgcucAggguuac.......................................................................  41      1  seq
.....................uagguagucgUucuggguuac.......................................................................  5       1  seq
.....................uagguagucgcucCggguuac.......................................................................  37      1  seq
.....................Gagguagucgcucuggguuac.......................................................................  304     1  seq
.....................uagUuagucgcucuggguuac.......................................................................  7       1  seq
.....................uaggCagucgcucuggguuac.......................................................................  17      1  seq
.....................uagguagucgAucuggguuac.......................................................................  33      1  seq
.....................uagguagucgNucuggguuac.......................................................................  1       1  seq
.....................uagguagucCcucuggguuac.......................................................................  15      1  seq
.....................uagguagucgcucuggAuuac.......................................................................  15      1  seq
.....................Cagguagucgcucuggguuac.......................................................................  25      1  seq
.....................uagguaguAgcucuggguuac.......................................................................  45      1  seq
.....................uaAguagucgcucuggguuac.......................................................................  49      1  seq
.....................uagguagucgcucuUgguuac.......................................................................  13      1  seq
.....................uagguagAcgcucuggguuac.......................................................................  77      1  seq
.....................uagguNgucgcucuggguuac.......................................................................  10      1  seq
.....................uagguagucgGucuggguuac.......................................................................  11      1  seq
.....................uagguagucAcucuggguuac.......................................................................  33      1  seq
.....................uagguagucgcucuggCuuac.......................................................................  10      1  seq
.....................Nagguagucgcucuggguuac.......................................................................  43      1  seq
.....................uagAuagucgcucuggguuac.......................................................................  28      1  seq
.....................uaCguagucgcucuggguuac.......................................................................  7       1  seq
.....................uagguagucgcuGuggguuac.......................................................................  8       1  seq
.....................uagguagucgcucuAgguuac.......................................................................  224     1  seq
.....................uagguagucgcucuggguuaN.......................................................................  3       1  seq
.....................uagguaguUgcucuggguuac.......................................................................  45      1  seq
.....................Aagguagucgcucuggguuac.......................................................................  949     1  seq
.....................uagguagucgcucuggguuGc.......................................................................  15      1  seq
.....................uagguagucUcucuggguuac.......................................................................  14      1  seq
.....................uagguagucgcucuggguNac.......................................................................  1       1  seq
.....................uagguagCcgcucuggguuac.......................................................................  20      1  seq
.....................uagguagucgcucuguUguuac.......................................................................  23      1  seq
.....................uagguagucgcucugggAuac.......................................................................  53      1  seq
.....................uagguagucgcucuggNuuac.......................................................................  4       1  seq
.....................uagguagucgcucugggGuac.......................................................................  3       1  seq
.....................uagguagucgGucuggguuacu......................................................................  38      1  seq
.....................uagguagucgcuAuggguuacu......................................................................  192     1  seq
.....................uagguagucgcucGggguuacu......................................................................  46      1  seq
.....................uagguaAucgcucuggguuacu......................................................................  70      1  seq
.....................Aagguagucgcucuggguuacu......................................................................  3350    1  seq
.....................uagguagucgcucAggguuacu......................................................................  114     1  seq
.....................uUgguagucgcucuggguuacu......................................................................  65      1  seq
.....................uagguagucgcucugggCuacu......................................................................  72      1  seq
.....................uagguagucUcucuggguuacu......................................................................  48      1  seq
.....................uagguagucgcucugNguuacu......................................................................  1       1  seq
.....................uagguagucNcucuggguuacu......................................................................  9       1  seq
.....................uagguagucgcucuggAuuacu......................................................................  75      1  seq
.....................uagguagucgcucuggguuCcu......................................................................  8       1  seq
.....................uagguagucgcucugAguuacu......................................................................  444     1  seq
.....................uagguagNcgcucuggguuacu......................................................................  1       1  seq
.....................uagguagucgcucugggAuacu......................................................................  227     1  seq
.....................uagguagucgcucuggguuacu......................................................................  858902  0  seq
.....................uagUuagucgcucuggguuacu......................................................................  28      1  seq
.....................uagguagucgcCcuggguuacu......................................................................  219     1  seq
.....................uagguaguAgcucuggguuacu......................................................................  153     1  seq
.....................uagguCgucgcucuggguuacu......................................................................  5       1  seq
.....................uagguagucgcucuggguCacu......................................................................  99      1  seq
.....................uagguagucgcNcuggguuacu......................................................................  1       1  seq
.....................uagguagucgAucuggguuacu......................................................................  112     1  seq
.....................uaUguagucgcucuggguuacu......................................................................  43      1  seq
.....................uagguagucgcucuggguuaAu......................................................................  151     1  seq
.....................uagguagucgcucuggguuacG......................................................................  1285    1  seq
.....................uagguagucgcucuggguuGcu......................................................................  101     1  seq
.....................uagguagCcgcucuggguuacu......................................................................  71      1  seq
.....................uagguaCucgcucuggguuacu......................................................................  36      1  seq
.....................uagguagucgcucuggNuuacu......................................................................  1       1  seq
.....................uagguagucgcucuggguNcu.......................................................................  1       1  seq
.....................uagguUgucgcucuggguuacu......................................................................  78      1  seq
.....................uagguagAcgcucuggguuacu......................................................................  248     1  seq
```

```
                    Mature                                        Star
uguugcgcgggacccuucgauagguagucgcucuggguuacugucgggucguuauacccuuugcaaguggcucagggggacccccuauccaagggcugcuugaucgcuacgu
....................uagguaUucgcucuggguuacu......................................................................   39     1   seq
....................uagguagucgcucuggguGacu......................................................................   49     1   seq
....................uaCguagucgcucuggguuacu......................................................................   46     1   seq
....................uagguagucgcucuggguAacu......................................................................   94     1   seq
....................uagCuagucgcucuggguuacu......................................................................   30     1   seq
....................uagguagucgcuUuggguuacu......................................................................   163    1   seq
....................uagguagucgcucuggCuuacu......................................................................   37     1   seq
....................uagguagucgcucuggguuaNu......................................................................   7      1   seq
....................uagguagucAcucuggguuacu......................................................................   142    1   seq
....................uaNguagucgcucuggguuacu......................................................................   3      1   seq
....................uagguGgucgcucuggguuacu......................................................................   86     1   seq
....................uNgguagucgcucuggguuacu......................................................................   24     1   seq
....................uagguNgucgcucuggguuacu......................................................................   27     1   seq
....................uagguaguUgcucuggguuacu......................................................................   199    1   seq
....................uagguagucgcucCggguuacu......................................................................   109    1   seq
....................uagguagucgcucuggguuacC......................................................................   272    1   seq
....................uagguagucgcucuCgguuacu......................................................................   54     1   seq
....................uagguagGcgcucuggguuacu......................................................................   25     1   seq
....................uagguagucgcucNggguuacu......................................................................   5      1   seq
....................uaggGagucgcucuggguuacu......................................................................   108    1   seq
....................uCgguagucgcucuggguuacu......................................................................   11     1   seq
....................Cagguagucgcucuggguuacu......................................................................   121    1   seq
....................uagguagucgUucuggguuacu......................................................................   136    1   seq
....................uagguagucgcucuggguuacA......................................................................   2054   1   seq
....................uagguagucgcucuggguuacN......................................................................   3      1   seq
....................uaAguagucgcucuggguuacu......................................................................   219    1   seq
....................Gagguagucgcucuggguuacu......................................................................   1027   1   seq
....................uagguaguNgcucuggguuacu......................................................................   1      1   seq
....................uagguagucgcucugggGuacu......................................................................   71     1   seq
....................uagguagucgcucuAgguuacu......................................................................   1100   1   seq
....................uagguagucgcGcuggguuacu......................................................................   78     1   seq
....................uagguagucCcucuggguuacu......................................................................   34     1   seq
....................uagguagucgcucuggguuaGu......................................................................   45     1   seq
....................uagAuagucgcucuggguuacu......................................................................   79     1   seq
....................uagguaguGgcucuggguuacu......................................................................   89     1   seq
....................uagguagucgcucuUgguuacu......................................................................   42     1   seq
....................uagguagucgcucugUguuacu......................................................................   178    1   seq
....................uagguagucgcucuggguuUcu......................................................................   85     1   seq
....................uaggCagucgcucuggguuacu......................................................................   76     1   seq
....................uagguagucgcuGugggguuacu.....................................................................   51     1   seq
....................uGgguagucgcucuggguuacu......................................................................   238    1   seq
....................uagguagucgcAcuggguuacu......................................................................   118    1   seq
....................Nagguagucgcucuggguuacu......................................................................   227    1   seq
....................uagguagucgcucuggguNacu......................................................................   4      1   seq
....................uagguagucgcucuggUuuacu......................................................................   162    1   seq
....................uagguagucgcucuggguuaUu......................................................................   2531   1   seq
....................uagguagucgcucugCguuacu......................................................................   48     1   seq
....................uaggAagucgcucuggguuacu......................................................................   356    1   seq
....................uagguagucgcucuggguuacuN.....................................................................   4      1   seq
....................uagguagucgcucuggguuacuU.....................................................................   3456   1   seq
....................uagguagucgcucuggguuacug.....................................................................   3      0   seq
....................uagguagucgcucuggguuacuC.....................................................................   2      1   seq
....................uagguagucgcucuggguuacuA.....................................................................   1063   1   seq
....................uagguagucgcucuggguuacuAu....................................................................   1      1   seq
....................uagguagucgcucuggguuacuUu....................................................................   7      1   seq
.....................agguagucgcucuggguua........................................................................   70     0   seq
.....................Ugguagucgcucuggguua........................................................................   1      1   seq
.....................agguagucgcucuggguuac.......................................................................   498    0   seq
.....................Ggguagucgcucuggguuac.......................................................................   2      1   seq
.....................Ugguagucgcucuggguuac.......................................................................   1      1   seq
.....................agguagucgcucCggguuac.......................................................................   1      1   seq
.....................agguagucUcucuggguuacu......................................................................   1      1   seq
.....................agguagGcgcucuggguuacu......................................................................   1      1   seq
.....................agguagucgcAcuggguuacu......................................................................   1      1   seq
.....................agguagucgcucuggguuacu......................................................................   1591   0   seq
.....................Ugguagucgcucuggguuacu......................................................................   5      1   seq
.....................agguagucgcucuUgguuacu......................................................................   1      1   seq
.....................agguaNucgcucuggguuacu......................................................................   1      1   seq
.....................agguagucgAucuggguuacu......................................................................   1      1   seq
.....................agguagucgcucuAgguuacu......................................................................   2      1   seq
```

Mature Star

uguugcgcgggacccuucgauagguagucgcucuggguuacugucgggucguuauacccuuugcaaguggcucagggggacccccuaucaagggcugcuugaucgcuacgu			
.....................agguagucgcGcuggguuacu..	1	1	seq
.....................agguGgucgcucuggguuacu..	1	1	seq
.....................Ggguagucgcucuggguuacu..	4	1	seq
.....................agguagucgcucuggguuacG..	5	1	seq
.....................agguagucgcucuggguAacu..	1	1	seq
.....................agguaguUgcucuggguuacu..	1	1	seq
.....................agguagAcgcucuggguuacu..	1	1	seq
.....................agguagucgcuAuggguuacu..	1	1	seq
.....................agguagucgcucuggguuacuU...	131	1	seq
.....................agguagucgcucuggguuacug...	175	0	seq
......................gguagucgcucuggguuac...	1	0	seq
......................Uguagucgcucuggguuac...	2	1	seq
......................gguagucgcucuggguuacu..	78	0	seq
......................Uguagucgcucuggguuacu..	2	1	seq
.......................guagucgcucuggguuacu..	3	0	seq
........................uagucgcucuggguuacu..	2	0	seq
....aguggcucagggggacccccu..........................	53	0	seq
....guggcucagggggacccccuau........................	1	0	seq
....uggcucagggggacccccu..........................	18	0	seq
....uggcucagggggacccccuau........................	1	0	seq
....uggcucagggggacccccuauA.......................	1	1	seq
....uggcucaAggggacccccuauc.......................	1	1	seq
....uggcucagggAgacccccuauc.......................	1	1	seq
....uggcucagggggacccccuauc.......................	245	0	seq
....uggcucaggAggacccccuauc.......................	3	1	seq
....uggcucagggggacccccuauU.......................	1	1	seq
....uggcucagggggacccccuaucU......................	1	1	seq
....uggcucagggggacccccuaucc......................	5	0	seq
....ggcucagggggacccccuaucc......................	1	0	seq
